# Fungal Warriors: Effects of *Beauveria bassiana* and *Purpureocillium lilacinum* on CCYV-Carrying Whiteflies

**DOI:** 10.3390/biom15040593

**Published:** 2025-04-16

**Authors:** Dan Zhai, Hang Lu, Suyao Liu, Jialei Liu, Wanyu Zhang, Jingjing Wu, Jingjing Li, Rune Bai, Fengming Yan, Chenchen Zhao

**Affiliations:** Henan International Laboratory for Green Pest Control, Henan Engineering Laboratory of Pest Biological Control, College of Plant Protection, Henan Agricultural University, Zhengzhou 450046, China; z13598789312@163.com (D.Z.); lhang_990912@163.com (H.L.); 17629601980@163.com (S.L.); liujialei2022@163.com (J.L.); 18236368710@163.com (W.Z.); wujingjing559901@163.com (J.W.); jjli@henau.edu.cn (J.L.); yxbre@163.com (R.B.)

**Keywords:** biological control, insect–fungal interactions, non-viruliferous versus viruliferous whiteflies, symbionts, bio-pesticides

## Abstract

*Bemisia tabaci* is a major agricultural pest that affects both greenhouse and field crops by feeding on plant sap, which impairs plant growth, and by secreting honeydew, promotes sooty mold growth that further reduces photosynthesis. Additionally, these insects are vectors for viruses such as the cucurbit chlorotic yellows virus (CCYV), which causes significant damage to cucurbit crops. Traditional chemical pesticide treatments have limitations, including the development of resistance, harm to non-target organisms, and environmental contamination. Traditional chemical pesticides have limitations when it comes to controlling plants infested by CCYV and whitefly. However, the underlying reasons for these limitations remain unclear, as does the impact of entomopathogenic fungi on whitefly responses. This study explores the potential of using biological control agents, specifically *Beauveria bassiana* and *Purpureocillium lilacinum*, to manage whitefly populations and control CCYV transmission. Laboratory experiments were conducted to evaluate the pathogenicity of these fungi on non/viruliferous whitefly. The results indicated that both fungi effectively reduced whitefly populations, with *B. bassiana* showing particularly strong adverse effects. Whiteflies infected with CCYV exhibited a higher LC_50_ to *B. bassiana* and *P. lilacinum*. Furthermore, bio-pesticides significantly altered the bacterial microbiome dynamics of the whitefly. Interestingly, CCYV increased the susceptibility of whiteflies to entomopathogenic fungus. The findings suggest that these biocontrol agents offer a sustainable alternative to chemical pesticides. Our study unraveled a new horizon for the multiple interaction theories among bio-pesticides–insects–symbionts–viruses.

## 1. Introduction

*Bemisia tabaci*, a pervasive agricultural pest commonly known as whitefly, poses a substantial threat to both greenhouse and field crop production globally [1]. The insect primarily infests solanaceous, cucurbitaceous, and leguminous crops by feeding on plant sap, which directly stunts growth. Additionally, their excretion of honeydew promotes the production of sooty mold, further impeding photosynthetic capabilities and reducing crop yields [2,3,4]. Beyond direct damage, whiteflies vector numerous plant viruses, notably the cucurbit chlorotic yellows virus (CCYV), which they transmit in a semi-persistent manner [5]. CCYV particularly affects cucurbit crops like melons and cucumbers, causing leaf yellowing and stunting that significantly impacts harvest quality and quantity [6].

Conventional whitefly management has traditionally relied on chemical pesticides [7]. However, this approach presents several challenges: increasing pesticide resistance in whitefly populations, causing harm to beneficial insects and pollinators, and resulting in potential environmental and food contamination [8]. These limitations highlight the need for integrated pest management (IPM) strategies [9,10].

Virus-infected whiteflies present additional management challenges due to their enhanced insecticide resistance [11]. This resistance develops through physiological adaptations that reduce pesticide effectiveness, including the improved metabolic breakdown of chemicals and alterations to insecticide target sites [12,13]. Resistance development is accelerated by whiteflies’ genetic diversity and high reproductive rates, which rapidly spread resistant traits through populations [14,15]. Evidence suggests that viral infection stress may trigger the increased expression of resistance genes as part of their survival mechanisms [16]. Effectively managing insecticide resistance in virus-infected whiteflies requires an integrated approach combining chemical controls with biological controls, cultural practices, and resistant plant varieties [17]. This IPM strategy reduces insecticide dependence, slowing resistance development while maintaining effective control options [11].

Biological control represents a sustainable, environmentally friendly alternative within IPM frameworks. By utilizing the natural enemies of whiteflies—predators, parasitoids, and pathogens—this approach maintains pest populations at manageable levels without chemical treatment side effects. This aligns with efforts to reduce agricultural chemical use while supporting biodiversity and ecosystem health [18]. Entomopathogenic fungi (EPF) show particular promise as biological control agents, infecting insects by penetrating their cuticle and disrupting both gut microbiota and immune responses [19].

*Beauveria bassiana* and *Purpureocillium lilacinum* are naturally occurring fungi affecting many insect species, representing promising control agents [20]. Upon contact, the fungus penetrates the insect’s exoskeleton and proliferates inside the body, causing the insect to die, typically within days [21]. The use of *B. bassiana* in controlling whiteflies capitalizes on its high virulence and specificity to the pests while being safe for non-target organisms, including humans. This makes it an ideal candidate for integration into IPM programs, especially in greenhouse environments where whiteflies can be particularly problematic [22].

Given the challenges presented by *B. tabaci*, CCYV-infected populations, and chemical control limitations, biological control options merit further investigation [17]. Among these, *B. bassiana* and *P. lilacinum* stand out for their specific insect host interactions and minimal non-target impacts.

We propose a comprehensive study assessing *B. bassiana* and *P. lilacinum* pathogenicity against CCYV-infected whiteflies. This research will evaluate these fungi’s effectiveness in reducing whitefly populations and disrupting CCYV transmission cycles. Additionally, we will explore the broader ecological impacts of introducing these biocontrol agents, particularly their effects on whitefly microbial communities. Our approach involves first assessing the baseline toxicity of *B. bassiana* and *P. lilacinum* to whiteflies in laboratory settings, followed by the analysis of whitefly microbial community dynamics after EPF exposure.

This approach not only aims to provide a sustainable solution to the whitefly problem but also contributes to a healthier agricultural environment by reducing chemical inputs and supporting biodiversity. Our findings could significantly advance integrated pest management strategies, offering a more holistic and environmentally friendly approach to agricultural pest management.

## 2. Materials and Methods

### 2.1. Plants, Virus, Fungi, and Insects

As outlined in our previous work, we acquired plants, insects, and viruses following the methodologies detailed therein [16]. The whiteflies were housed under controlled laboratory conditions, also described in our earlier research [16], with rearing parameters set at 26 ± 1 °C, a relative humidity of 65 ± 5%, and a photoperiod of 16 h of light and 8 h of darkness. The cryptic species *B. tabaci* Mediterranean (MED; biotype Q) was cultivated on cucumber plants at Henan Agricultural University for over six years under separate maintenance protocols.

Cucumber (*Cucumis sativus* L. cv. Bojie-107) and cotton (*Gossypium hirsutum* L. cv. ICR-49) plants were cultivated in a greenhouse. The plants were separately potted in containers of 10 cm in diameter and 12 cm in height, situated within specialized insect-proof cages measuring 60 × 40 × 80 cm. Cucumber and cotton seedlings were selected for the experimental methods when reaching the 3–4 true leaf developmental stage. All cucumber plants were assessed for CCYV infection before the commencement of the trial.

CCYV plants were crucial for both maintaining the viral clone and conducting transmission assays. Cotton serves as a suitable host for the whitefly, while it does not support CCYV infection. Consequently, cotton was utilized to evaluate the duration for which whiteflies could transmit the virus after their removal from CCYV-infected hosts.

The experimental procedure involved initially allowing the CCYV-bearing whiteflies to feed on infected plants, followed by their transfer to healthy, non-infected cucumber plants, thus facilitating effective virus transmission. Our findings revealed that non-viruliferous adult whiteflies were capable of acquiring CCYV upon feeding on infected plants [23].

The virulence status of whiteflies and plants were determined through quantitative real-time PCR using specific primers targeting the coat protein coding sequence of CCYV (GenBank Accession No. QVG59408) [23,24], with the forward primer spanning nucleotide positions 548–567 (5′-GCGACCATCATCTACAGGCA-3′) and the reverse primer targeting nucleotide positions 679–699 (5′-CCGACTTGTTCCTTTCAGAGC-3′). qRT-PCR was performed using TB Green Premix Ex Taq^TM^ II (Takara, Code No. RR820A) in a 20 µL reaction system containing 10 µL of the qPCR master mix, 1 µL of the cDNA template or plasmid dilution, 0.8 µL of 10 µM of each primer, 0.4 µL of ROX Reference Dye II, and 7 µL of nuclease-free water. The amplification program was as follows: initial denaturation at 94 °C for 2 min, followed by 40 cycles of denaturation at 94 °C for 15 s, annealing at 60 °C for 20 s, and extension at 72 °C for 20 s. The virulence titers in each sample were calculated by interpolating the Ct values against the standard curve, providing a reliable measure of virulence levels. Furthermore, we managed to maintain colonies of both non-viruliferous and viruliferous whiteflies on cucumber and cotton plants across more than 50 generations, without any exposure to insecticides.

The entomopathogenic fungi *Beauveria bassiana* were isolated from *Bombyx mori* in the wild in Shenyang city, Liaoning Province, China. *Purpureocillium lilacinum* was kindly provided by Professor Long Liu of Henan Agricultural University.

Both fungal strains showed virulence to whiteflies. They underwent rigorous taxonomic confirmation through multi-locus sequence analysis (MLSA) targeting the ITS, TEF-1α, and RPB2 loci, and were subsequently maintained on potato dextrose agar (PDA) under standardized culture conditions (25 °C, 12/12 h light/dark cycle, 75% RH) with biweekly sub-culturing, after which they were preserved in 20% glycerol at −80 °C.

**Figure 1:**
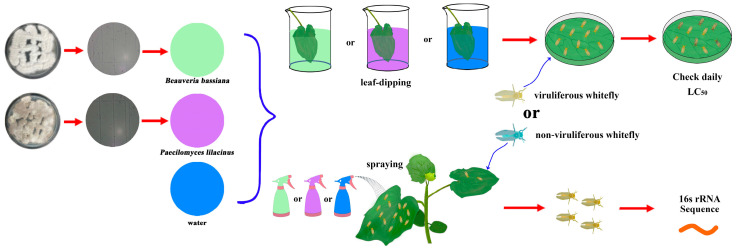
Bioassay and effects of *B. bassiana* and *P. lilacinum* on whiteflies.

The colonies of *B. bassiana* exhibited spreading filamentous growth with white pigmentation on the obverse surface and pale coloration on the reverse (Figure 1). These fungal colonies of *P. lilacinum* exhibited spreading filamentous growth with irregular margins, presenting opaque white obverse surfaces featuring rugose striations and pale-yellow reverse pigmentation, characterized by a planar morphology, desiccated texture, and cerebriform folding (Figure 1).

Both fungal isolates were successively sub-cultured for five generations on the PDA medium at 25 °C (*B. bassiana*) and 28 °C (*P. lilacinum*) with 7-day transfer intervals. Subsequently, the conidia were harvested using a 0.05% Tween 80 aqueous solution and then stored at 4 °C. This storage method ensured their viability for a duration of three to four weeks. For the purposes of our experiments, we selected only those conidia that demonstrated a germination rate above 90%, ensuring the consistency and reliability of our results.

### 2.2. Entomopathogenic Fungi Bioassay of Non-Viruliferous and Viruliferous Whiteflies

The bioassays of *B. bassiana* and *P. lilacinum* were conducted as described in previous studies, which was by the leaf dip method [17], containing 100 μL of the conidial suspension (10^7^ to 10^3^ of *B. bassiana* and 10^8^ to 10^3^ of *P. lilacinum*) or sterilized tap water (with 0.05% Tween 80, control) to soak for 10 s, respectively. Three-day-old adults of non-viruliferous and viruliferous whiteflies were chosen for the experiment.

Whitefly mortality was assessed at 48 h by quantifying the deceased insects to calculate LC_50_ (the concentration needed to kill 50% of the adults (Figure 1)). Each concentration was replicated five times, with each biological replicate containing 20 individual whiteflies. Biological replicates were conducted on separate days using whiteflies from distinct rearing batches to minimize batch-specific variability. Individuals of the same generation for every replicate were randomly assigned to the treatment or control groups via a random number table, and mortality was assessed by two independent researchers blinded to group identities. Whiteflies were considered dead when they did not react for at least 5 min, and the body turned black after being touched with a soft brush. We also used pathogenicity verification for the survival status and the reasons for the death of whiteflies.

### 2.3. Effect of B. bassiana and P. lilacinum on the Symbiotic Bacteria to Non-Viruliferous and Viruliferous Whiteflies

The effects of *B. bassiana* and *P. lilacinum* on symbiotic bacteria were investigated and analyzed as previously described [17]. Three-day-old adult non-viruliferous and viruliferous whiteflies were separately introduced to cucumber plants. The cucumber plants were individually treated with the LC_50_ of non-viruliferous and viruliferous whiteflies of *B. bassiana* and *P. lilacinum*, separately. The solution was uniformly applied to the cucumber leaves using a manual sprayer, and 0.05% Tween 80 was sprayed as a control (Figure 1). The samples were obtained from whiteflies exposed to *B. bassiana*- or *P. lilacinum*-infected and uninfected diets 24 h post-feeding. Whitefly adults were collected rapidly, frozen in liquid nitrogen, and preserved at −80 °C for subsequent analysis (DNA extraction). We tested each concentration in five replicates, with each replicate consisting of >100 individual insects.

The microbiota composition of whitefly individuals was characterized using MiSeq sequencing targeting the V3–V4 region of the bacterial 16S rRNA gene. Samples were obtained from whiteflies exposed to *B. bassiana*- or *P. lilacinum*-infected and uninfected diets 24 h post-feeding. Genomic DNA was extracted from these samples utilizing the MolPure Cell/Tissue DNA Kit (Yeasen Biotechnology, Shanghai Co., Ltd., Shanghai, China) in accordance with the manufacturer’s protocol. To ensure the removal of surface contaminants, the whiteflies were surface-sterilized by triple rinsing in 75% ethanol followed by sterile water prior to DNA extraction. A lysozyme treatment (50 mg/mL, Vazyme Biotech Co., Ltd., Nanjing, China) was applied to facilitate the lysis of Gram-positive bacterial cells, with incubation at 37 °C for 30 min. DNA quality and quantity were assessed using a NanoDrop 2000C spectrophotometer (Thermo Scientific, Waltham, MA, USA) and agarose gel electrophoresis. The V3–V4 region of the 16S rRNA gene was amplified using the 338F/806R primer pair [19]. Subsequent procedures, including PCR product handling, library construction, and sequencing, were carried out according to established protocols [20]. Sequencing was performed on an Illumina MiSeq platform (Illumina, San Diego, CA, USA), with purified amplicons pooled in equimolar concentrations for paired-end sequencing. The raw sequencing data have been deposited in NCBI under BioProject accession number PRJNA1019092.

### 2.4. Quantification of Bacteria Communities

To detect the presence of *Portiera*, *Rickettsia*, *Hamiltonella*, and *Cardinium* in whiteflies, the adults were homogenized in lysis buffer and DNA was extracted and amplified using the protocol described. The specific primers used for detection were amplified according to previous studies (Table S1) [25]. qPCR was employed to quantify the copies of 16S rRNA genes relative to the total DNA copies, following an established protocol as described in the previous study [26]. To validate primer specificity, each primer was initially designed to target variable regions within the 16S rRNA gene sequences of interest. Primer-BLAST was employed to assess the specificity of each primer pair individually, ensuring targeted amplification without cross-reactivity. Specificity was further confirmed by testing each primer pair against standard vectors, where no non-target amplification was observed. Additionally, melt curve analysis of qPCR products indicated that each primer pair generated a single distinct amplification product, confirming their specificity and suitability for downstream applications.

### 2.5. Bioinformatics and Statistical Analyses

The LC_50_ values were calculated using probit analysis in IBM SPSS Statistics 20.0, with the results expressed as mean ± standard error (SE). Statistical comparisons of bacterial populations were performed using one-way ANOVA with LSD’s post hoc test with a significant level set at 0.05 (SAS Institute Inc.,Cary, NC, USA), supplemented by Kruskal–Wallis tests for non-normal distributions.

Bioinformatics analyses were performed on the Majorbio I-Sanger Cloud Platform (https://www.sanger.ac.uk/, accessed on 1 September 2024). The microbial community was delineated into operational taxonomic units (OTUs) at a 97% similarity threshold and analyzed via Uparse software (v7.0.1001, http://drive5.com/uparse/, accessed on 1 September 2024). To address discrepancies in read depth among samples, alpha diversity analyses (mothur, version v.1.30.2, https://mothur.org/wiki/calculators/, accessed on 1 September 2024), were conducted using data standardized through rarefaction to the lowest sequence depth (1,921,738 reads). Metrics for alpha diversity included observed species richness, ACE and Chao1 estimators, Simpson and Shannon indices, and Good’s coverage, providing a comprehensive assessment of within-sample diversity. Beta diversity (*R*, version 3.3.1) was examined through principal coordinate analysis (PCoA) and analysis of similarity (ANOSIM, vegan, version 2.4.3), both calculated with Bray–Curtis distances. Comparisons were further refined by examining treated whiteflies and normal whiteflies, using PERMANOVA with both weighted and unweighted UniFrac distances to capture between-sample diversity patterns (with false discovery rate, FDR, correction).

## 3. Results

### 3.1. B. bassiana and P. lilacinum Pathogenicity Against Whiteflies

**Figure 2:**
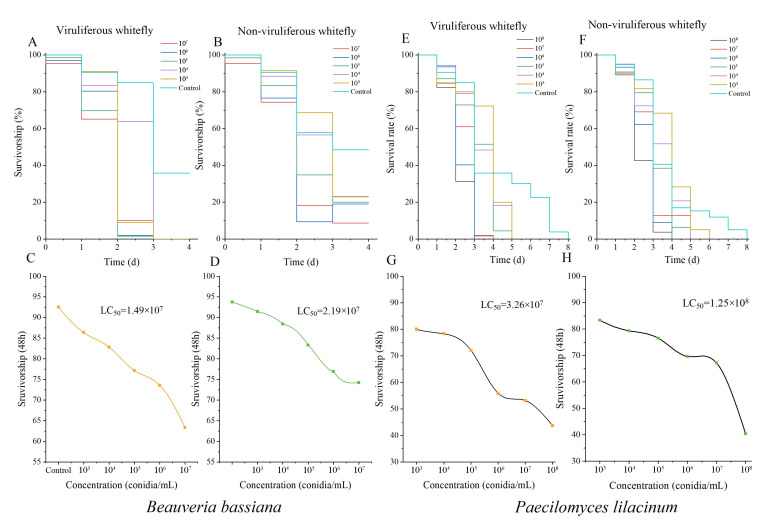
Direct toxicity and LC50 effects of *B. bassiana* and *P. lilacinum* at various concentrations on whitefly survival in feeding bioassays. Panels (**A**,**E**) illustrate the survival rates of non-viruliferous whiteflies exposed to *B. bassiana* and *P. lilacinum*, respectively, while panels (**B**,**F**) show the survival rates for viruliferous whiteflies. Panels (**C**,**G**) display the LC_50_ values of *B. bassiana* and *P. lilacinum* for non-viruliferous whiteflies, and panels (**D**,**H**) represent the LC50 values for viruliferous whiteflies. Each data point reflects the mean of five biological replicates ± SEM. For EPF, the graphs depict fitted values based on quadratic logistic regression. LC_50_ refers to the concentration needed to achieve 50% mortality after 48 h of exposure.

To assess the pathogenicity of *B. bassiana* and *P. lilacinum* on whiteflies, we examined the susceptibility of fungus-exposed whiteflies. Our findings revealed that viruliferous whiteflies displayed significantly higher susceptibility to fungal infection compared to non-viruliferous whiteflies. In the control group, however, viruliferous whiteflies exhibited higher viability than their non-viruliferous counterparts (Figure 2). Additionally, *B. bassiana* demonstrated superior insecticidal efficacy compared to *P. lilacinum*.

### 3.2. Overview of Microbiotas in Whiteflies Fed on B. bassiana and P. lilacinum

To investigate whether *B. bassiana* and *P. lilacinum* influence the whitefly microbiota composition, we exposed whiteflies to various LC_50_ concentrations of each fungus separately. We characterized the microbial communities in treated whiteflies using 16S rRNA sequencing. The analysis yielded 1,807,850 sequences, 752,795,828 bp, and 184 OTUs (phylum: 16, class: 21, order: 54, family: 88, genus: 135, species: 165) with an average read length of 416 bp. Good’s coverage estimates indicated that over 99% of the species diversity was captured in the 30 samples (Table S2), signifying that the sampling depth was adequate. Rarefaction curves, approaching a saturation plateau, revealed notable variability in OTU numbers across different samples, with a higher density of OTUs observed in the upper layers compared to the lower strata. Additionally, the data indicated that *B. bassiana* and *P. lilacinum* influenced the richness of the whitefly community (Figure S1).

### 3.3. Effect of B. bassiana and P. lilacinum on Microbiome Diversity in Whiteflies

**Figure 3:**
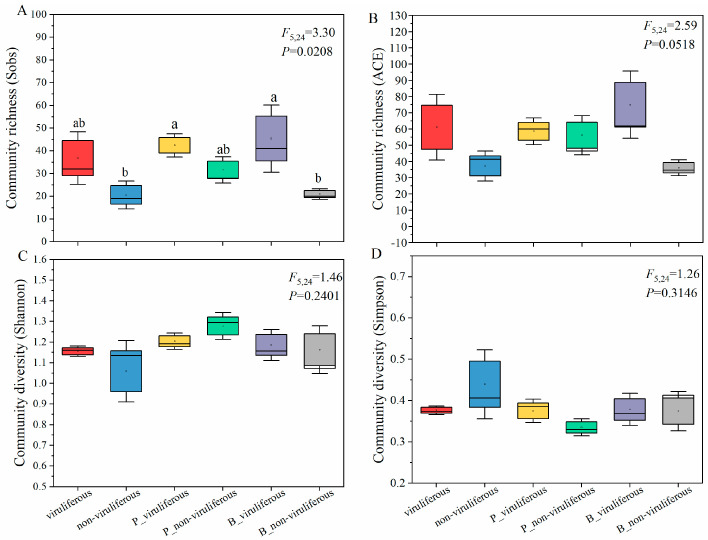
Boxplot of species richness and community diversity of the bacterial communities in whiteflies after EPF exposure. Alpha diversity plots measured with Obs (**A**), ACE (**B**), Shannon (**C**), and Simpson (**D**) indices of samples. Different lowercase labels above each group indicate significant differences (one-way ANOVA, LSD post hoc test, *p* < 0.05) of group mean values.

We compared the bacterial communities between non-viruliferous and viruliferous whiteflies, focusing specifically on how exposure to *P. lilacinum* and *B. bassiana* affected microbiome composition. Alpha diversity metrics revealed that after EPF treatment, bacterial richness (measured by ACE and observed species indices) was significantly higher in viruliferous whiteflies compared to non-viruliferous whiteflies. In contrast, microbial diversity (as assessed by the Shannon and Simpson indices) showed a slight reduction, although these differences were not statistically significant (Figure 3, Table S1). In the control group, bacterial richness, measured by both the ACE and Sobs indices as well as Shannon diversity, was notably higher in viruliferous whiteflies compared to non-viruliferous whiteflies (Figure 3A,C). However, following EPF exposure, viruliferous whiteflies only exhibited modest, statistically non-significant increases in both richness and diversity. Notably, CCYV presence in the control groups appeared to stimulate microbial richness and diversity in whiteflies (Figure 3A,C), following a trend similar to that observed in fungal treatments.

Non-viruliferous whiteflies showed a lower average variation degree (AVD) value than viruliferous whiteflies (Figure S2), which indicated higher microbiome stability. *P. lilacinum/B. bassiana* could increase the microbiome stability of viruliferous whiteflies but decrease the microbiome stability of non-viruliferous whiteflies (Figure S2). Additionally, as microbial diversity decreased in whiteflies, the AVD of the microbial community increased, resulting in reduced stability.

Taken together, these results indicate that exposure to *P. lilacinum*/*B. bassiana* and CCYV infection can impact the alpha diversity metrics of the whitefly microbiota. While notable differences were observed between viruliferous and non-viruliferous groups, the changes within each group after fungal exposure were generally minor and often not statistically significant. Further research is needed to better understand the biological implications of these subtle variations.

**Figure 4:**
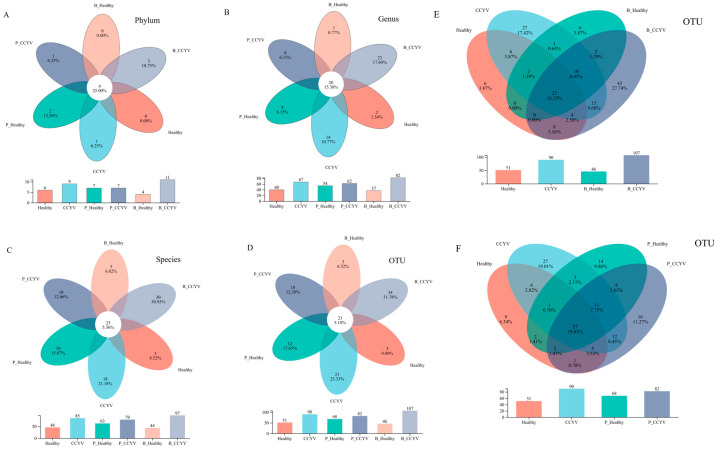
Venn diagrams depicting the overlap of the bacterial community in the non-viruliferous and viruliferous whiteflies after exposure to *P. lilacinum*/*B. bassiana* at phylum (**A**), genus (**B**), species (**C**), and OTU (**D**–**F**) levels. Notes: CCYV means viruliferous whiteflies, healthy means non-viruliferous whiteflies, B means *B. bassiana* exposure, and P means *P. lilacinum* exposure.

To compare the microbial community composition between treatment groups, we generated Venn diagrams illustrating shared bacterial taxa (Figure 4). Across all six whitefly groups, we identified 4 phyla, 20 genera, 23 species, and 23 OTUs. There were 5 and 21 core OTUs confined to the control non/viruliferous whitefly groups, respectively (Figure 4). Viruliferous whiteflies harbored more species than non-viruliferous whiteflies. Interestingly, *P. lilacinum* could increase the OTU numbers of non-viruliferous whiteflies (12), but decrease the OTU numbers of viruliferous whiteflies (10). *B. bassiana* presented the opposite situation (Figure 4). Additionally, there were 6, 27, 6, and 43 core OTUs confined to each of the non/viruliferous whiteflies and to when they were exposed to *B. bassiana*, respectively (Figure 4E), with a total of 25 core OTUs common to all groups. When exposed to *P. lilacinum*, the core OTUs were 9, 27, 14, and 16, with a total of 27 core OTUs being common to all groups (Figure 4F). This suggests that EPF could regulate community composition and showed opposite effects on non/viruliferous whiteflies, especially when the viruliferous whiteflies were exposed to *B. bassiana.*

**Figure 5:**
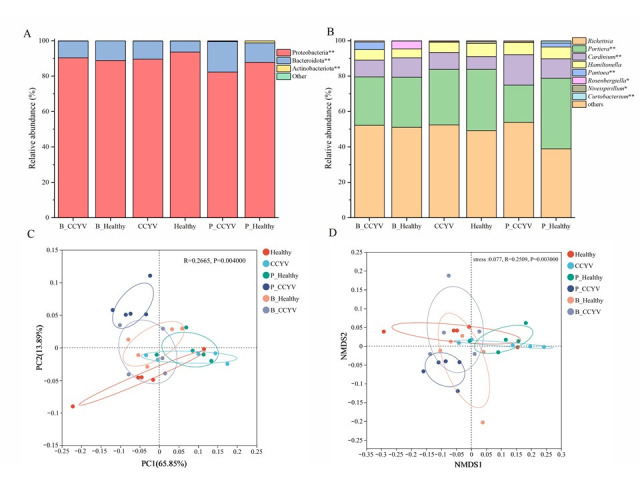
Bacterial community composition in non-viruliferous and viruliferous whiteflies following exposure to *P. lilacinum* and *B. bassiana*. (**A**) presents the bacterial composition at the phylum level, while (**B**) shows the composition at the genus level. Statistical significance is indicated using a non-parametric Kruskal–Wallis test with LSD’s post hoc test (*: 0.01 < *p* ≤ 0.05, **: 0.001 < *p* ≤ 0.01). (**C**) Principal coordinate analysis (PCoA) and (**D**) non-metric multidimensional scaling (NMDS) based on Bray–Curtis dissimilarities are included to illustrate the bacterial community structure. The explained variance for each axis in the PCoA and NMDS is provided in parentheses.

Taxonomic analysis across all samples revealed Proteobacteria as the dominant phylum, followed by Bacteroidota and Actinobacteriota (Figure 5A). We observed significant differences in bacterial composition between non-viruliferous and viruliferous whiteflies, with notable variations in the relative abundance of bacterial taxa among the treatment groups. Specifically, Proteobacteria exhibited the highest relative abundance in the control group of non-viruliferous whiteflies (93.60%) and in the viruliferous whiteflies exposed to *B. bassiana* (90.38%). However, a substantial decline was observed in the viruliferous whitefly group (89.62%), with a further reduction upon exposure to *P. lilacinum* (82.36%). Bacteroidota, which constituted 6.31% of the bacterial community in the control group of non-viruliferous whiteflies, exhibited a marked increase in viruliferous whiteflies (10.25%). This trend was further accentuated in viruliferous whiteflies exposed to *P. lilacinum* (17.18%), while exposure to *B. bassiana* did not result in substantial changes (9.47%). In non-viruliferous whiteflies, exposure to *P. lilacinum* and *B. bassiana* resulted in Bacteroidota proportions of 10.98% and 11.00%, respectively. The relative abundance of Actinobacteriota was notably low in the control group (0.06%) but exhibited an increase upon exposure to *P. lilacinum* (0.27% in viruliferous whiteflies and 1.12% in non-viruliferous whiteflies). Conversely, *B. bassiana* exposure led to a slight reduction in Actinobacteriota in non-viruliferous whiteflies (0.04%) but an increase in viruliferous whiteflies (0.08%). These findings suggest that fungal exposure and viral status play a pivotal role in modulating the bacterial community composition of whiteflies, potentially influencing their microbiome dynamics and associated ecological functions.

A heatmap illustrating the distribution of the 35 most abundant genera provided comprehensive insight into the bacterial community structure (Figure S3). At the genus level (Figure 5B), Rickettsia was the most predominant phylum, contributing around 50% of the bacteria in all whitefly groups, except non-viruliferous whiteflies exposed to *P. lilacinum* (28.92%). Portiera emerged as the second most predominant bacterial genus within the whitefly population (31.33% in viruliferous whiteflies, 34.55% in non-viruliferous whiteflies). Its abundance decreased after being exposed to EPF (<28.32%), except in non-viruliferous whiteflies exposed to *P. lilacinum*, where it increased (39.90%). Notably, viruliferous whiteflies exposed to *P. lilacinum* contained a higher proportion of Cardinium (17.18%) than the other treatments (<11.03%). Hamiltonella also constituted a principal microbial component of the control group in non-/viruliferous whiteflies (6.22%, 7.01%) but comprised a lesser portion of *B. bassiana*-exposed whiteflies (5.08%, 5.89%). It showed no significant difference in *P. lilacinum*-exposed whiteflies (6.74%, 7.00%). The whiteflies generally lacked the presence of Pantoea, but otherwise harbored a higher portion when viruliferous whiteflies were exposed to *B. bassiana* (4.36%) and non-viruliferous whiteflies were exposed to *P. lilacinum* (2.14%). It is interesting that Rosenbergiella was absent in *P. lilacinum*-treated whiteflies and the control group of non-viruliferous whiteflies, and had a lower abundance in viruliferous whiteflies (<0.02%) but showed a higher abundance in *B. bassiana*-treated non-viruliferous whiteflies (Figure 5B).

We analyzed beta diversity through cluster tree analysis, principal coordinate analysis (PCoA), and non-metric multidimensional scaling (NMDS). Cluster analysis indicated a clear separation between the *B. bassiana* and *P. lilacinum* exposure groups, with greater similarity within the same treatment groups (Figure S4). PCoA based on OTUs revealed a separation of the non-viruliferous whitefly and viruliferous whiteflies exposed to EPF, with the first two principal components accounting for 65.85% and 13.89% of the total variation, suggesting that EPF exposure significantly influenced community structure (Figure 5C). NMDS analysis also demonstrated a clear separation between groups (Figure 5D).

We used Linear Discriminant Analysis Effect Size (LEfSe) to identify significantly enriched taxa (LDA scores > 4) within treatment groups (Figures S5 and S6). In the control non-viruliferous whiteflies, few bacteria were significantly enriched, namely Proteobacteria. In the viruliferous whiteflies, four groups of bacteria were detected to be significantly enriched, namely Gammaproteobacteria, Oceanospirillales, Halomonadaceae, and Portiera (Figures S5 and S6). In the viruliferous whiteflies exposed to *P. lilacinum*, we detected the significant enrichment of multiple bacteria, including Bacteroidota, Amoebophilaceae, Cytophagales, Cardinium, and Bacteroidia. *B. bassiana* exposure resulted in fewer significantly enriched microbes: Erwiniaceae was enriched in non-viruliferous whiteflies, while Pantoea was significantly enriched in viruliferous whiteflies (Figures S5 and S6). These shifts in microbial community composition appeared to be closely linked with variations in EPF exposure and CCYV presence, suggesting that EPF play a crucial role in shaping microbial community structure in whitefly populations.

### 3.4. Effect of B. bassiana and P. lilacinum on Gene Experssion in Whiteflies

The qPCR verification demonstrated that the abundance of four key bacterial symbionts (*Portiera*, *Rickettsia*, *Hamiltonella*, and *Cardinium*) in whiteflies remained largely unaffected by treatment with EPF, suggesting a limited effect of fungal treatment on these bacteria in general. However, EPF treatment had differing effects depending on the viral status of the whiteflies. In viruliferous whiteflies, EPF suppressed the abundance of *Portiera*, *Rickettsia*, and *Cardinium*, whereas in non-viruliferous whiteflies, it stimulated their populations.

**Figure 6:**
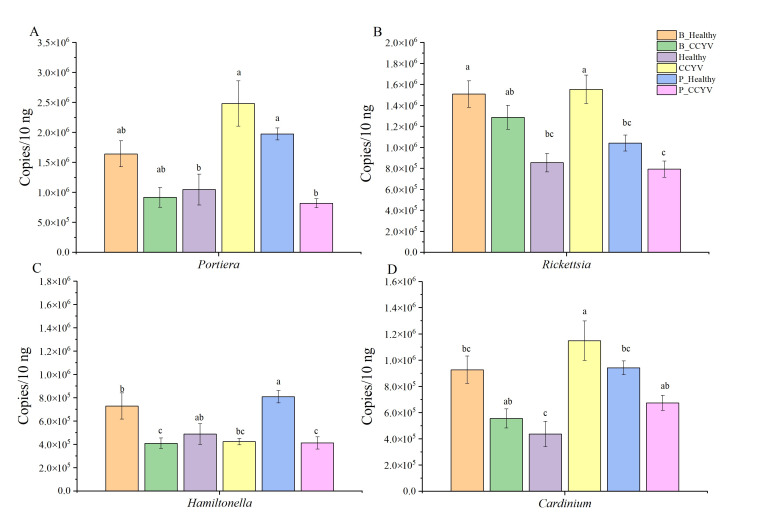
Bacterial numbers of 16S rRNA gene copies of dominant bacterial species (**A** *Portiera*, **B** *Rickettsia*, **C** *Hamiltonella*, and **D** *Cardinium*) in whiteflies fed *B. bassiana* and *P. lilacinum*. Statistical significance calculated based on one-way ANOVA test. Different lowercase labels above each group indicate significant differences (one-way ANOVA, LSD post hoc test, *p* < 0.05) of group mean values.

Notably, the type of fungal species used influenced bacterial copy numbers. For instance, whiteflies exposed to *B. bassiana* exhibited higher bacterial copy numbers of *Portiera* and *Rickettsia* compared to those treated with *P. lilacinum*, suggesting a potential species-specific interaction between the fungi and the whitefly microbiota. Additionally, treatment with CCYV significantly affected the symbiotic bacterial populations. Specifically, *Portiera*, *Rickettsia*, and *Cardinium* showed increased abundance, while *Hamiltonella* was suppressed. These findings suggest that CCYV modulates the whitefly microbiome by selectively enhancing certain bacterial populations while diminishing others (Figure 6).

Interestingly, the impact of entomopathogenic fungi on bacterial copy numbers appeared to be influenced by the viral status of the host. In viruliferous whiteflies, fungal treatments resulted in a decrease in bacterial abundance, which may have reflected a shift in the host’s immune response or microbial environment due to the presence of the virus. In contrast, fungal treatments in non-viruliferous whiteflies led to an increase in the copy numbers of these bacteria, suggesting that the absence of viral infection may have facilitated a more favorable environment for bacterial growth.

## 4. Discussion

Plant viruses often enhance vector attraction to infected hosts but exhibit varying effects on vector behavior, particularly in terms of settling, feeding preferences, and overall performance. Persistent transmission (PT) viruses generally improve the host plant’s suitability for vectors, thereby facilitating prolonged feeding. In contrast, non-persistent transmission (NPT) viruses tend to diminish plant quality, encouraging the rapid dispersal of vectors after brief feeding periods. These divergent strategies reflect the distinct evolutionary pressures faced by different types of plant viruses, with PT viruses promoting sustained interaction with the host and NPT viruses favoring quick vector movement to maximize their spread [27]. Viruses have evolved mechanisms to enhance their transmission via insect vectors. After infecting plants, they suppress the plant’s immune response, facilitating insect feeding and increasing insect reproduction, which in turn raises the likelihood of virus spread [28,29]. Furthermore, some viruses alter the feeding behavior of infected insects, causing them to preferentially feed on infected or healthy plants. This behavioral modification significantly enhances the potential for viral transmission, contributing to the broader dispersal of the virus across plant populations [30,31]. Control of virus vectors has been a most effective means is to prevent the transmission of plant viruses, but has remained a global challenge [32]. Therefore, in this experiment, we checked the population fitness of whiteflies infected by NPT viruses (CCYV) under EPF.

In this study, we identified several important findings: (1) entomopathogenic fungi (EPF) demonstrate strong pathogenicity, with viruliferous whiteflies showing significantly higher sensitivity compared to non-viruliferous whiteflies; (2) EPF negatively affects the microbial community structure within whiteflies; and (3) alterations in core symbionts influence the sensitivity of viruliferous whiteflies to EPF.

Previous studies have demonstrated that non-viruliferous whiteflies exhibit a higher preference for virus-infected plants, while viruliferous whiteflies are more inclined to choose uninfected plants. This behavior significantly facilitates the spread of the virus, as it enhances viral acquisition by non-viruliferous whiteflies and promotes viral inoculation on uninfected plants by viruliferous whiteflies [33]. This increases the difficulty of prevention. Current strategies for preventing and managing plant virus outbreaks largely rely on targeting the associated vectors. As a result, insecticides are pivotal in safeguarding global food security. Nonetheless, excessive insecticide application in agricultural settings has driven the development of resistance in insect vectors, compromising the effectiveness of these measures [15]. Interestingly, numerous studies have documented correlations between virus transmission and insecticide resistance [34]. Whiteflies infected with the persistently transmitted tomato yellow leaf curl virus (TYLCV) showed increased resistance to the insecticide Flupyradifurone [35]. Foliar application of pyrifluquinazon and afidopyropen reduced the tomato chlorosis virus (ToCV) transmission and significantly decreased the ToCV loads in tomato plants [34]. Phytoplasma induced a leaf color change to attract insect vectors [36]. “Trade-off” between virus transmission and insecticide resistance in a vector is an interesting topic in the context of coevolution.

Entomopathogenic fungi like *B. bassiana* provide a sustainable alternative to chemical insecticides, uniquely infecting hosts by penetrating their exoskeletons. This makes them especially effective against sap-sucking pests. [37]. The EPF *B. bassiana* and *M. anisopliae* have demonstrated significant potential as effective biocontrol agents against whitefly pests. These fungi exhibit remarkable pathogenicity, offering a promising alternative to chemical pesticides in integrated pest management strategies. Their ability to infect and kill whiteflies, coupled with their environmental sustainability, positions them as a valuable tool in the management of whitefly populations, contributing to the reduction in pest-induced damage while minimizing the ecological impact of conventional control methods [38]. EPF are vital biological control agents, widely used to manage insect pests [39]. Sequential applications of EPF show promise for sustainable Bemisia tabaci nymph control [40]. However, limited research exists on their performance against *B. tabaci* and CCYV transmission. Interestingly, previous research indicated that whiteflies exhibit significant resistance to insecticides [12]. Our findings indicate that CCYV-infected whiteflies are more susceptible to EPF than non-infected ones. Notably, CCYV enhances whitefly resilience to insecticides [16], potentially by modulating apoptosis and autophagy to maintain viral stability [41]. This susceptibility shift suggests that EPF may disrupt this immune balance, offering a novel means to reduce viral transmission.

A symbiont-focused management strategy could offer an effective alternative for delaying resistance development in whiteflies and assisting with pesticide remediation in agricultural fields [42]. Coevolution with endosymbionts enables sap-feeding insects to thrive [43]. Gut microbes are a potential target for insecticide development [44].Fungal infections induce dysbiosis in the mosquito gut microbiome by significantly increasing the abundance of specific bacteria while reducing microbial diversity, ultimately contributing to mosquito mortality [45]. A previous study demonstrated that *M. anisopliae* produces metabolites that activate immune responses and suppress the locust gut microbiota, highlighting a fungal strategy to manipulate host immunity and microbiota during infection [19]. Previous research has demonstrated that symbionts influence both the infection and transmission of plant viruses [46]. Exploring these interactions offers valuable insights into interspecies microbial relationships throughout the animal kingdom.

In the current work, we explored microbial community dynamics that were closely associated with variations in EPF exposure and CCYV infection. Bacterial composition significantly differed between whiteflies exposed to *B. bassiana* and *P. lilacinum*, yet within each group, the bacterial communities showed greater similarity. This suggests that EPF play a key role in shaping microbial community structures in whitefly populations. Non-viruliferous whiteflies displayed the highest microbiome stability, which may explain their reduced sensitivity to EPF compared to viruliferous individuals. EPF appear to modulate bacterial diversity, particularly in viruliferous whiteflies. Communities with higher phylogenetic diversity were more stable, indicating that microbiomes with greater biodiversity are more resilient to disturbances [47]. This could elucidate why viruliferous whiteflies exhibited heightened sensitivity to EPF.

In our study, *Portiera*, *Rickettsia*, and *Cardinium* were upregulated in viruliferous whiteflies, while CCYV suppressed *Hamiltonella* expression. The relationship between symbionts and insecticides varies widely [48]. The facultative symbiont *Rickettsia* has strong links to virus transmission [49], such as cotton leaf curl Multan virus (CLCuMuV) [50] and TYLCV [51]. Symbiotic bacteria can influence plant defenses, enhancing SA-regulated defenses while suppressing JA-regulated ones. Though Rickettsia previously made plants more resistant to *Verticillium dahliae* [25], in our study, it increased whitefly sensitivity to EPF. CCYV likely induced *Rickettsia*, and *Rickettsia*-infected whiteflies, often drawn to virus-infected plants, became more vulnerable to EPF. Prior studies also linked Rickettsia to higher whitefly susceptibility to thiamethoxam and related compounds [52,53].

*Hamiltonella* mediates whitefly–plant interactions by suppressing induced plant defenses [54]. In wheat aphids, *Hamiltonella* enhances insecticide tolerance [55,56]. However, in whiteflies, CCYV reduces *Hamiltonella* abundance, while non-viruliferous whiteflies exhibit higher levels of *Hamiltonella*, conferring resistance to EPF. Primary symbionts, such as *Candidatus Portiera*, are essential for host survival, while secondary symbionts have less consistent impacts [57]. All whiteflies carry *Portiera* within specialized bacteriocytes [58]. Elevated *Portiera* levels have been linked to increased whitefly susceptibility to thiamethoxam [48], and, in our study, to EPF in viruliferous whiteflies. Additionally, *Cardinium* suppresses plant defenses, reduces whitefly detoxification, and impairs probing behavior [59,60]. Its abundance in viruliferous whiteflies increased their sensitivity to EPF.

Our findings highlight the heightened susceptibility of viruliferous whiteflies to EPF, emphasizing the role of symbionts in whitefly–virus interactions. This provides valuable insights for leveraging symbionts to control virus transmission and improve pest management [61].Recent advancements in cross-kingdom RNAi offer a promising avenue for improving the effectiveness of entomopathogenic fungi against mosquitoes and other insect pests [37,62]. Our future experiments will focus on engineering fungal strains to produce host-specific miRNAs, aiming to reduce the risk of resistance development in target insect populations.

## 5. Conclusions

In conclusion, this study demonstrates the significant potential of entomopathogenic fungi (EPF), particularly *B. bassiana* and *M. anisopliae*, as eco-friendly biological control agents against *B. tabaci*. Our findings reveal enhanced fungal pathogenicity against viruliferous whiteflies, suggesting EPF’s effectiveness in managing CCYV transmission. Importantly, viruliferous whiteflies show increased susceptibility to EPF, likely due to CCYV-induced alterations in symbiont profiles.

The study highlights how symbiotic bacteria modulate whitefly responses to EPF treatment, with notable shifts in *Rickettsia*, *Portiera*, and *Cardinium* abundance in viruliferous whiteflies. These findings illuminate the complex interactions between host microbiota and fungal pathogenicity, offering valuable insights for enhancing biocontrol strategies. Our research provides a foundation for integrating EPF into sustainable pest management frameworks, reducing the dependence on chemical pesticides while addressing resistance concerns. Future research should explore advanced approaches such as cross-kingdom RNAi to engineer fungal strains targeting specific symbionts or host pathways, potentially improving biocontrol efficacy while minimizing resistance development. Moreover, by promoting biodiversity and reducing chemical inputs, EPF application aligns with global sustainable agriculture goals, representing an important component of integrated pest management systems for combating agricultural pests and associated diseases.

## Data Availability

The raw sequencing data have been deposited in NCBI under BioProject accession number PRJNA1019092.

## Supplementary Materials

The following supporting information can be downloaded at: https://www.mdpi.com/article/10.3390/biom15040593/s1, Table S1: PCR primers for *Portiera*, *Rickettsia*, *Hamiltonella*, and *Cardinium* detection in whiteflies. Table S2: Analysis of sequencing data from 30 whitefly samples across different developmental treatments. Figure S1: Rarefaction analysis of bacterial diversity in whitefly samples. Figure S2: Bacterial community variation across whitefly samples represented by average variation degree (AVD). Figure S3: Genus-level taxonomic heatmap visualizing the relative abundances of predominant bacterial taxa across whitefly samples. Color intensity corresponds to the abundance levels of each bacterial genus. Figure S4: Hierarchical clustering dendrogram constructed using the Unweighted Pair-Group Method with Arithmetic means (UPGMA) showing the relationships between bacterial communities in whiteflies based on 16S rRNA gene sequence data. Figures S5 and S6: Taxonomic cladogram generated through Linear Discriminant Analysis Effect Size (LEfSe) highlighting the bacterial taxa significantly enriched in whiteflies. Only taxa with LDA scores exceeding 4 are displayed.
