# Supplementary material for: Fungal Warriors: Effects of Beauveria bassiana and Purpureocillium lilacinum on CCYV-Carrying Whiteflies
Source: Biomolecules. 2025 Apr 16;15(4):593. doi: 10.3390/biom15040593 (PMC12025157; doi:10.3390/biom15040593)
Supplement: Supplementary file 1 [file biomolecules-15-00593-s001.zip › biomolecules-3550914-supplementary.pdf]

Table S1. PCR primers for *Portiera*、*Rickettsia*、*Hamiltonella*、*Cardinium* detection in whitefly.

Primer	Sequence (5'-3')
Portiera-F	AAGAAGCACCGGCTAACTCC
Portiera-R	ACCTACATGCCCTTTACGCC
Rickettsia-F	ACCGCCTACGCACTCTTTAC
Rickettsia-R	GCCTGATCCAGCAATACCGA
Hamiltonella-F	GGTAATACGGAGGGTGCGAG
Hamiltonella-R	CCAAGTTGAGCTCGGGGATT
Cardinium-F	CACTGAGATACGGGCCAGAC
Cardinium-R	CGCAGGGATTGTTTTGCTCC

Table S2. Analysis of sequencing data from 30 whitefly samples across different developmental treatments.The table presents key metrics including read count statistics, operational taxonomic unit (OTU) richness at 97% similarity threshold, Good's coverage estimates, alpha diversity in-dices (Shannon, ACE, Simpson), and species richness estimators (Sobs, Chao1) to characterize microbial community diversity and composition.

**Sample**	**Seq_num**	**Base_num**	**avg**	**min**	max	Alpha diversity estimators					
						Sobs	ACE	Chao	Shannon	Simpson	coverage
**CCYV_1**	**72624**	**30555274**	**421**	**398**	431	32	73.42	41.10	1.168	0.365	99.96%
CCYV_2	47660	19772235	415	363	433	23	39.63	30.00	1.094	0.401	99.98%
CCYV_3	40989	17289668	422	377	430	32	36.59	33.36	1.160	0.381	99.98%
CCYV_4	47231	19803853	419	377	435	30	47.53	41.14	1.157	0.362	99.97%
CCYV_5	49452	20571001	416	212	431	67	108.44	97.00	1.198	0.373	99.94%
HEALTHY_1	69066	28719606	416	203	435	20	41.35	27.00	1.151	0.382	99.98%
HEALTHY_2	51962	21251861	409	210	435	16	19.58	17.50	0.675	0.655	99.99%
HEALTHY_3	75983	31888720	420	202	435	12	44.32	22.00	1.236	0.337	99.99%
HEALTHY_4	57581	23871555	415	254	435	36	54.03	42.43	1.135	0.406	99.97%
HEALTHY_5	59548	24665545	414	278	435	19	27.10	26.00	1.097	0.417	99.98%
Q_CCYV_1	41045	17055586	416	278	430	52	62.74	61.75	1.252	0.352	99.97%
Q_CCYV_2	43157	17883112	414	203	435	37	44.97	43.43	1.159	0.386	99.97%
Q_CCYV_3	43966	18199472	414	262	469	49	59.94	53.71	1.192	0.393	99.97%
Q_CCYV_4	40649	16780848	413	277	430	39	49.00	43.09	1.139	0.427	99.97%
Q_CCYV_5	67171	27982759	417	363	433	35	76.09	61.25	1.275	0.317	99.96%
Q_HEALTHY_1	51367	21541363	419	356	435	35	46.48	41.88	1.295	0.330	99.97%
Q_HEALTHY_2	68703	28557885	416	262	435	26	39.99	37.00	1.135	0.385	99.97%
Q_HEALTHY_3	59051	24711203	418	285	439	45	67.17	55.00	1.250	0.331	99.96%
Q_HEALTHY_4	65010	27335756	420	363	435	24	82.42	35.00	1.401	0.301	99.97%
Q_HEALTHY_5	69495	29097516	419	203	435	28	45.00	47.50	1.308	0.328	99.97%
B_CCYV_1	93284	38956276	418	239	435	30	61.56	56.25	1.345	0.310	99.96%
B_CCYV_2	65215	26944959	413	230	465	83	106.45	100.55	1.137	0.431	99.93%
B_CCYV_3	59319	24538501	414	360	432	44	107.49	72.50	1.049	0.445	99.95%
B_CCYV_4	41841	17439026	417	203	463	29	37.58	33.67	1.156	0.368	99.98%
B_CCYV_5	46513	19381174	417	236	434	41	61.78	56.00	1.243	0.339	99.96%
B_HEALTHY_1	99552	41232279	414	237	435	18	34.67	30.00	1.028	0.427	99.98%
B_HEALTHY_2	67220	27793734	413	245	433	26	31.42	31.25	1.073	0.430	99.98%

B_HEALTHY_3	78665	32947521	419	262	454	18	48.69	25.00	1.438	0.268	99.98%
B_HEALTHY_4	84343	35197125	417	233	430	23	31.05	28.14	1.225	0.335	99.98%
B_HEALTHY_5	50188	20830415	415	363	435	20	34.99	25.25	1.051	0.412	99.98%

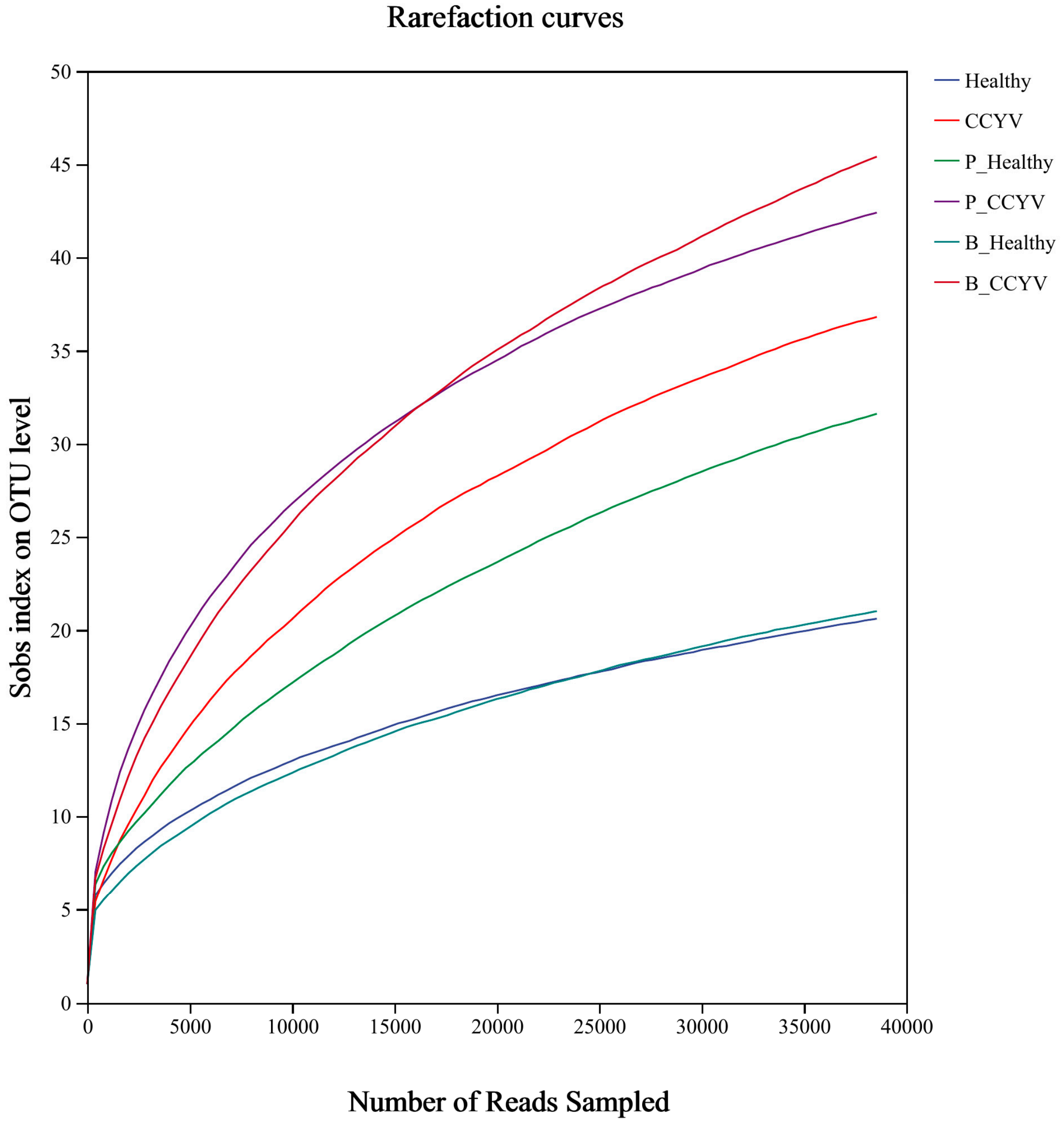

Figure S1. Rarefaction analysis of bacterial diversity in whitefly samples.

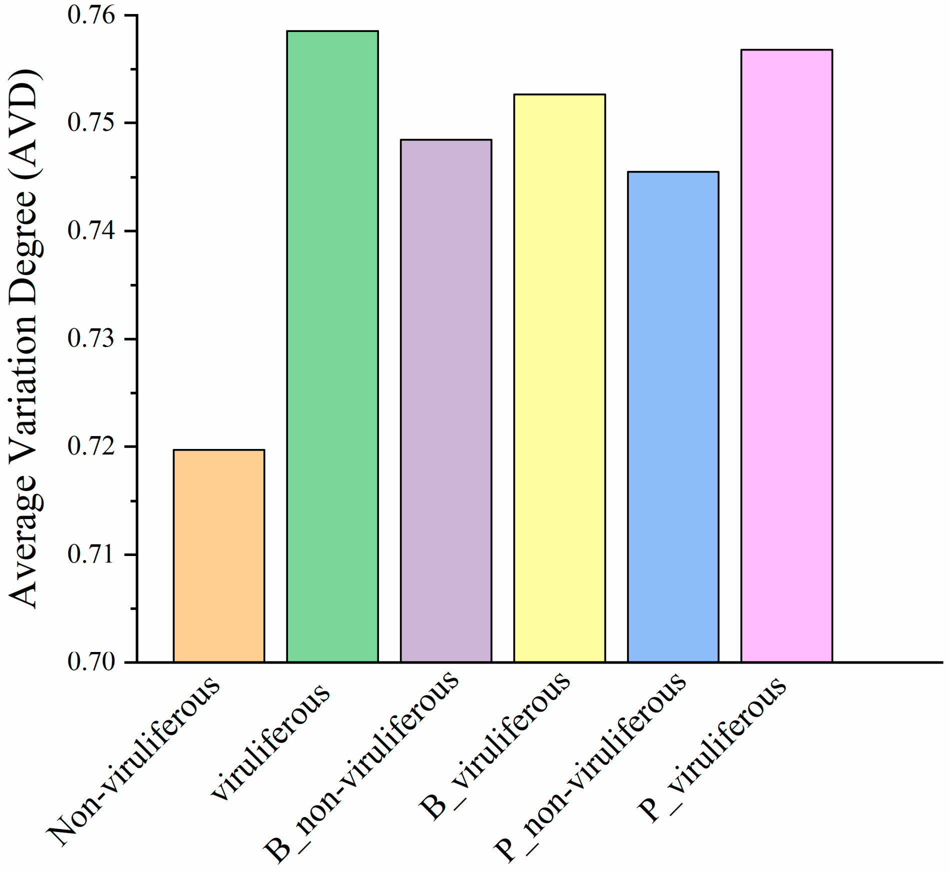

.

Figure S2. Bacterial community variation across whitefly samples represented by Average Variation Degree (AVD).

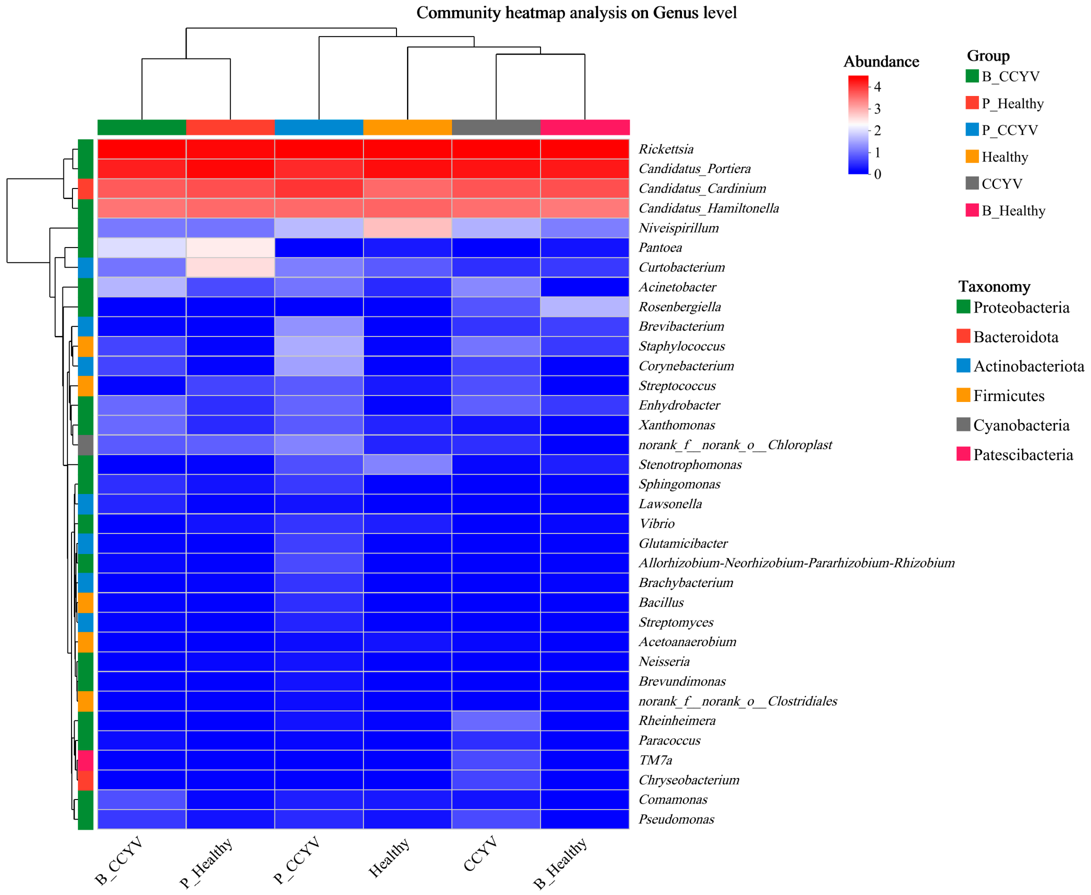

Figure S3. Genus-level taxonomic heatmap visualizing the relative abundance of predominant bacterial taxa across whitefly samples. Color intensity corresponds to abundance levels of each bacterial genus.

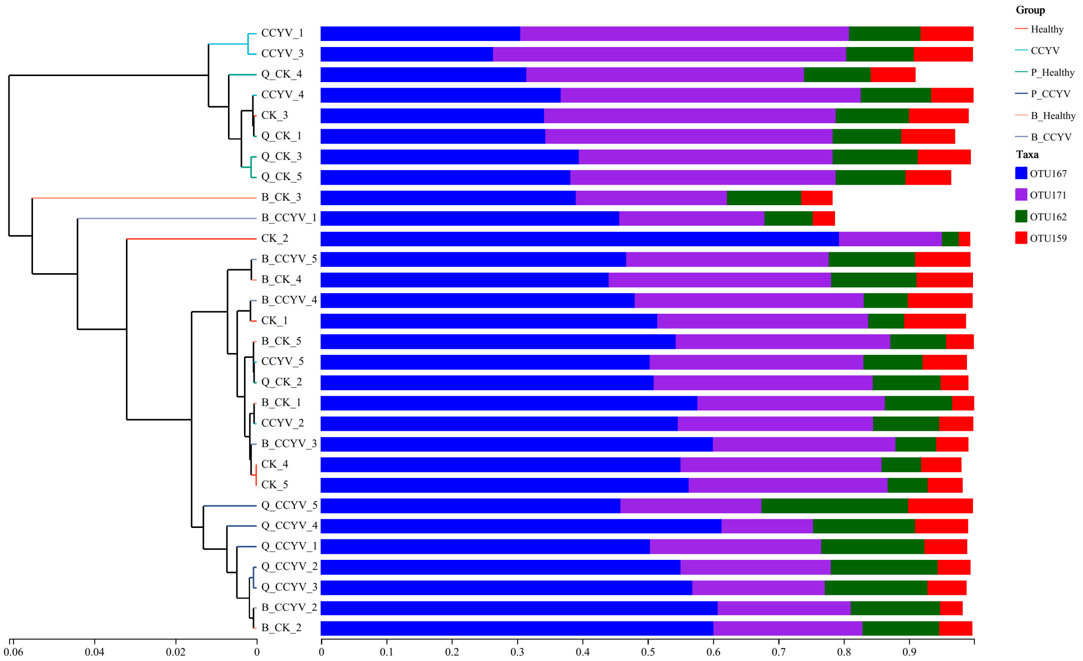

Figure S4. Hierarchical clustering dendrogram constructed using the Unweighted Pair-Group Method with Arithmetic means (UPGMA) showing relationships between bacterial communities in whitefly based on 16S rRNA gene sequence data.

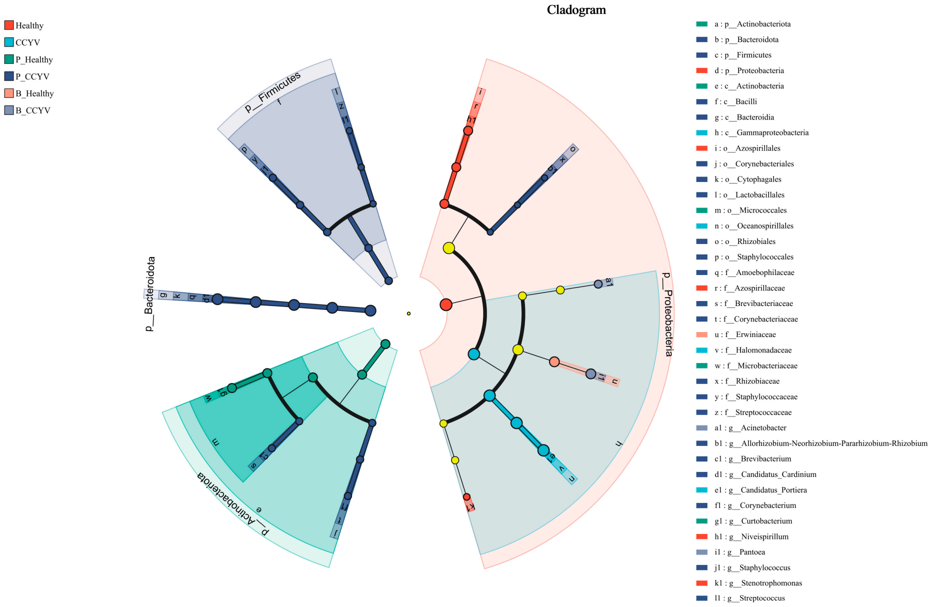

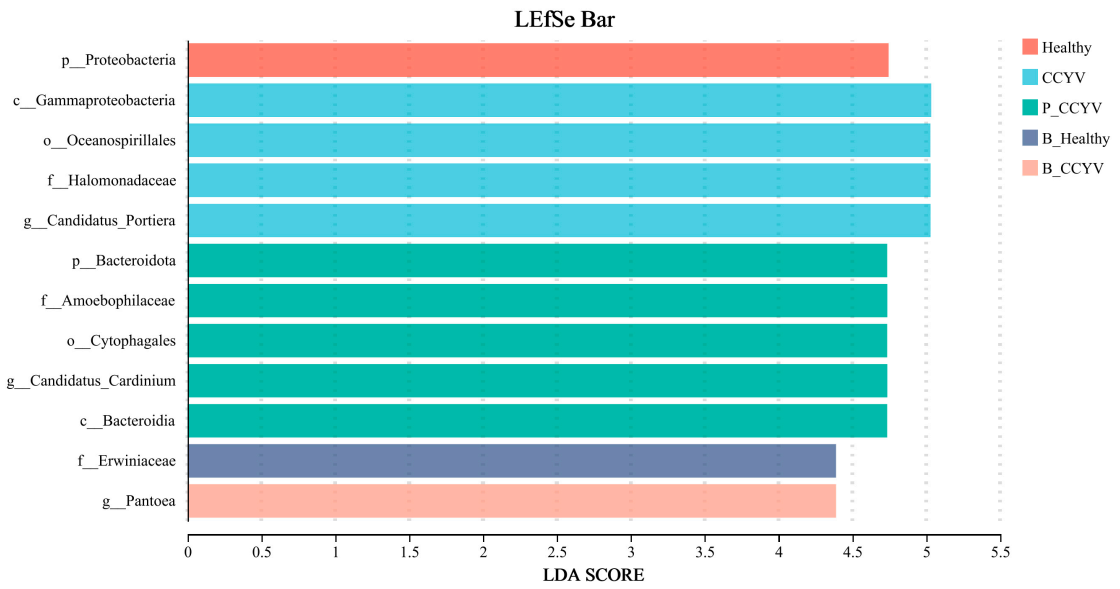

Figure S5. and S6: Taxonomic cladogram generated through Linear Discriminant Analysis Effect Size (LEfSe) highlighting bacterial taxa significantly enriched in whitefly. Only taxa with LDA scores exceeding 4 are displayed.
